# A Vegetarian-Style Dietary Pattern Is Associated with Lower Energy, Saturated Fat, and Sodium Intakes; and Higher Whole Grains, Legumes, Nuts, and Soy Intakes by Adults: National Health and Nutrition Examination Surveys 2013–2016

**DOI:** 10.3390/nu12092668

**Published:** 2020-09-01

**Authors:** Shanthy A Bowman

**Affiliations:** Beltsville Human Nutrition Research Center, Agricultural Research Service, U.S. Department of Agriculture, 10300 Baltimore Ave, Beltsville, MD 20705, USA; shanthy.bowman@usda.gov; Tel.: +1-301-504-0619

**Keywords:** Dietary Guidelines for Americans, vegetarian diet, nutrients, food pattern groups, NHANES, adults

## Abstract

Consumer demand for plant-based foods is increasing though the reasons may vary. Plant foods are sole sources of dietary fiber, vitamin C, and flavonoids and good sources of vitamin B1, folic acid, potassium, and magnesium. They are low in saturated fat, and do not contain cholesterol and vitamin B12. Plant foods are associated with better body weight and healthy blood lipid profile. This cross-sectional study used nationally representative National Health and Nutrition Examination Survey 2013–2016 data and compared nutrient and food pattern food group intakes of adults eating a vegetarian-style diet with adults eating a nonvegetarian diet. Adults 20+ years (*N* = 10,064) were grouped using the Dietary Guidelines for Americans 2015–2020 definition of vegetarian-style diet, which is modelled as lacto-ovo-vegetarian diet. Trained dietary interviewers collected self-reported dietary intake data using a 24-h recall and an automated multi-pass method. Means were compared using linear contrasts (*p* < 0.01). On average, the vegetarians ate an estimated 419 fewer kilocalories, 7 g less saturated fat, and 1274 mg less sodium. The vegetarian-style diet was higher in micronutrient density, except for vitamin B12 and zinc. The vegetarians ate more whole grains, legumes, nuts, and soy products. Fruit, vegetables, and dairy intakes were similar for both groups. A vegetarian-style diet may be advocated to control energy, saturated fat, and sodium intakes.

## 1. Introduction

There is a growing trend among consumers for clean, natural, and sustainable foods, which has increased demand for plant-based foods and alternate food products [1]. Plant-based proteins and meat-alternates are one of the top trends in the restaurant industry [2]. The National Restaurant Association research predicts that plant-based protein food products will continue to grow in popularity during the next decade [3]. In the recent years, clean meat or cultured meat is gaining popularity because it can be produced commercially using cell culture in controlled environment, without having to rear or slaughter animals [4]. 

In general, vegetarian diet includes plant-based foods and excludes all animal foods. However, the definition of vegetarian diet varies in the United States. Vegan diet includes plant-based foods and excludes all animal foods such as meat, poultry, seafood, dairy, and eggs. Lacto-ovo-vegetarian diet includes dairy and eggs in addition to plant-based foods. Pesco-Vegetarian diet includes seafood, dairy, eggs, in addition to plant-based foods. The Dietary Guidelines for Americans (DGA), which form the basis for the Federal nutrition policy and programs recommend that Americans practice a healthy eating pattern by increasing their vegetables, fruit, whole grain intakes and limit added sugars, saturated fat, and sodium [5,6]. The DGA 2015–2020 provides examples of a few healthy eating patterns including, a healthy vegetarian-style eating pattern. The healthy vegetarian-style dietary pattern as defined by the Dietary Guidelines for Americans 2015–2020 is modelled as lacto-ovo-vegetarian style dietary pattern and includes plant-based foods, eggs, and dairy; and excludes meat, poultry, and seafood [5,6].

Plant foods are rich sources of many of the nutrients of public health concern [5,6]. They are nutrient-dense while being low in calories. They are the sole sources of dietary fiber, vitamin C, flavonoids; good sources of vitamin B1, folic acid, potassium, and magnesium; low in saturated fat; and not a source of cholesterol and vitamin B12. Plant foods are associated with better body weight control, better blood lipid profile, and promoted in the prevention of diet-related chronic diseases. [5,6].

A cross-sectional study using the nationally representative, Continuing Survey of Food Intakes by Individuals (CSFII) 1994–1996, found that vegetarian adults, 19 years and over, who did not eat meat, poultry, or seafood (lacto-ovo-vegetarians) on day-1 of the survey had lower energy, lower total fat, and lower saturated fat intakes than adults who reported eating meat, poultry, or seafood [7]. In addition, both vegetarian males and females had lower mean body mass index (BMI) than their respective counterparts [7]. Haddad and Tanzman, used the same cross-sectional CSFII 1994–1996 data, but classified adults as vegetarians or nonvegetarians, based on self-report [8]. Their study found that self-reported vegetarian adults, irrespective of whether they ate or did not eat meat, on the survey day, had a healthier dietary pattern than the nonvegetarians.

Several large-scale cohort studies have associated vegetarian dietary patterns with a reduced risk of diet-related chronic diseases. In the Adventists Health Study 2 (AHS-2), vegetarian dietary patterns have been associated with lower prevalence of obesity, hypertension, metabolic syndrome, and type 2 diabetes mellitus [9,10,11,12,13]. Other studies have reported a positive relationship between vegetarian diet and cardiovascular health [14,15]. In the European Prospective Investigation into Cancer and Nutrition (EPIC) study, vegetarians had a 32 percent lower risk of developing coronary heart disease, compared with nonvegetarians [16,17]. A review by Kahleova et al. found that plant-based diets were associated with decreased all-cause mortality and decreased risk of obesity, type 2 diabetes, and coronary heart disease [18]. Plant-based diets are associated with a reduced risk of colorectal cancer [19]. Wang et al. in a meta-analysis showed that vegetarian diets were associated with lower blood concentration of total cholesterol, low-density lipoprotein (LDL), and high-density lipoprotein cholesterol (HDL) as compared with other types of diet [20]. Similar association between vegetarian diets and blood lipid profile has been shown in a meta-analysis by Yokoyama et al. [21].

The studies discussed above used different types of validated instruments such as 24-h recall, food frequency questionnaires, and/or food diary to collect dietary data; and also used different databases for nutrient estimations [7,22,23,24]. In spite of these methodology differences and a lack of uniform definition of a vegetarian diet, the study results collectively showed, a mostly plant-based dietary pattern helped prevent or reduce several diet-related chronic health conditions. Low calorie and high dietary fiber in the vegetarian diets are associated with lower BMI, which by itself is directly associated with several chronic conditions such as cardiovascular disease and hypertension. Low saturated fat and cholesterol are associated with better blood lipid profile, and low sodium with maintaining healthy blood pressure. However, vegetarian diet may also lack some of the essential micronutrients such as vitamin B12, calcium, iron, and zinc [25]. 

The current study differs from the other studies because it is nationally representative of the U.S. population at a specific survey period. This study classified foods into Dietary Guidelines defined, food pattern food groups, and portion sizes expressed in cup, ounce, and teaspoon equivalents [5,6]; and also used the DGA 2015–2020 definition to group adults into two groups: (1) Those who ate a vegetarian-style diet and (2) those who ate a nonvegetarian diet on the survey day. The DGA 2015–2020 form the basis for Federal nutrition policies and programs. Therefore, the study findings can be applied at population level and can be used for developing nutrition education and dietary intervention programs in the United States.

A priori objectives of the study were to identify the differences, if any, in the mean intakes of selected nutrients and food pattern food groups, body measurements, and serum cholesterol levels of adults eating a vegetarian-style diet and adults eating a nonvegetarian diet. 

## 2. Materials and Methods 

### 2.1. Definition of Vegetarian-Style Dietary Pattern

The healthy vegetarian-style dietary pattern as defined by the Dietary Guidelines for Americans 2015–2020, modelled as lacto-ovo-vegetarian diet, includes plant-based foods, eggs, and dairy; and excludes meat, poultry, and seafood [5,6]. The study used this DGA definition of vegetarian dietary pattern to categorize adults. 

### 2.2. Data Source and Sample

The study used cross-sectional data from National Health and Nutrition Examination Surveys (NHANES) conducted in 2013–2014 and 2015–2016 [26,27]. The NHANES is a program of studies designed to assess the health and nutritional status of adults and children in the United States and is conducted by the Centers for Disease Control and Prevention’s (CDC) National Center for Health Statistics (NCHS). The NHANES is nationally representative; and the data are released in two-year cycles. The survey examines a nationally representative sample of about 5000 persons each year. The demographic, body weight and blood lipid profile data were obtained from NHANES conducted in 2013–2016. The dietary data were obtained from the What We Eat in America (WWEIA) survey, the dietary component of NHANES 2013–2016. Trained dietary interviewers collected self-reported food and beverage intakes using a 24-h dietary recall and an automated multi-pass method (AMPM) [28]. The data files and details on the survey methodology are available on the Centers for Disease Control and Prevention Webpage [26,27]. Nutrient contents of each of the foods and beverages reported in the NHANES were computed using the Food and Nutrient Database for Dietary Studies (FNDDS) [29,30]. Each of the foods and beverages are then disaggregated into DGA defined food pattern food groups such as whole grains, refined grains, intact/whole fruit, 100% fruit juice, dark-green-, red-orange-, starchy-, and other vegetables, nuts and seeds, soy products, meat, poultry, and seafood, fluid milk, cheeses, yogurt, solid fats, oils, added sugars, and alcoholic drinks. There are 37 food pattern food groups in the Food Patterns Equivalents Database (FPED). As an example, in the FPED, sweetened chocolate milk will have fluid milk, added sugars, and solid fats food pattern food groups. The FNDDS and FPED are updated for each of the NHANES survey cycles and are specific for that survey periods. Readers may note the differences between food groups developed based on individual foods, and the food pattern food groups. For example, nuts and seeds food group developed using the individual foods in the survey, will contain raw and roasted nuts and seeds eaten as is. The nut and seeds food pattern food group in the FPED, in addition to the nuts and seeds eaten as is, will include nuts and seeds present in breakfast cereals, snack bars, candies, bakery desserts, dairy desserts, and other foods. The FPED data for the NHANES 2013–2016 and the methodology used to convert dietary intakes to Food Patterns equivalents is available at the Agricultural Research Service, Food Surveys Research Group Website [31,32,33]. 

Adults ages 20 years and over with complete, reliable dietary data on day 1 of WWEIA, NHANES 2013–2016 were included in the data analysis (*N* = 10,064). The DGA 2015–2020 definition of vegetarian-style dietary pattern was used to divide the adults into two groups: (1) adults who had a DGA defined vegetarian-style dietary pattern (also referred as vegetarians in this study) and (2) adults who did not have a vegetarian-style dietary pattern (nonvegetarians). 

### 2.3. Data Analysis

The following were analyzed: (1) Percentages of adults who reported eating a vegetarian-style diet by sex, race-ethnicity, and percentage poverty level, which is the ratio of family income to poverty expressed as a percentage (% poverty level). Odds ratios for eating a vegetarian-style diet were estimated using logistic regression models adjusting for sex (male, female), race-ethnicity (non-Hispanic white, non-Hispanic black, non-Hispanic Asian, Hispanic), and percentage poverty level poverty level (<131%, 131% to 350%, over 350% poverty level) as categorical variables, and age as a continuous variable. (2) Unadjusted mean energy and selected nutrient and food pattern food group intakes; and mean intakes adjusted for sex, race-ethnicity, % poverty level, and age as a continuous variable in the linear regression models. (3) Nutrient density of selected macro- and micronutrients per 1000 kilocalories of energy intakes. (4) Mean body mass index (BMI), waist circumference, and percentages of overweight males and females (BMI 25 or higher), and (5) mean serum total cholesterol and serum high-density lipoprotein cholesterol. More than half the sample did not have serum low-density lipoprotein (LDL)-cholesterol and triglycerides data, and hence, these two blood lipid profiles were included in the data analysis, but not reported.

Data were analyzed using SAS 9.4 (SAS Institute, Cary, NC, USA) and SAS-Callable SUDAAN release 11.0.3 (Research Triangle Institute, Research Triangle Park, NC, USA) statistical software. The data analyses used survey sample weights and design effects. Means were compared using linear contrasts. A *p*-value < 0.01 was considered significantly different for all adjusted and unadjusted mean comparisons.

## 3. Results

### 3.1. Demographic Characteristics

Table 1 includes the percentages of adults and the odds ratios for eating a vegetarian-style diet by demographic characteristics. Overall seven percent of all adults ate a vegetarian-style diet, as defined by the dietary guidelines. More females (8.4%) than males (5.5%) ate a vegetarian-style diet; and females had higher odds of eating a vegetarian-style diet. Among the race-ethnic groups, a significantly high percentage of non-Hispanic Asians (14.5%) reported eating a vegetarian-style diet, followed by non-Hispanic white (7.1%) and Hispanics (6.7%) adults. Only a small percentage of non-Hispanic blacks (3.4%) reported eating a vegetarian-style diet. Non-Hispanic Asians are twice as likely and non-Hispanic blacks are half as likely as non-Hispanic white to eat a vegetarian-style diet. No significant differences were noted among the percent poverty level groups.

### 3.2. Nutrient Intakes

Unadjusted mean intakes of adults eating a vegetarian-style diet were lower in energy, fat, and protein and higher in dietary fiber than the adults who ate a nonvegetarian diet (Table 2). 

The vegetarians ate 419 kcals less energy, 19 g less total fat, 7 g less saturated fat, 140 mg less cholesterol, and 33 g less protein. After adjusting for the demographic characteristics, these differences continued to be significant. The differences in the adjusted means were 335 kcals energy, 16 g total fat, 6 g saturated fat, 128 mg cholesterol, and 31 g protein. 

Moreover, the vegetarian-style diet was significantly lower in some essential micronutrients. The differences between the two groups were: 0.5 mg vitamin B6, 1.9 µg vitamin B12, 0.9 µg vitamin D, 275 mg phosphorus, 381 mg potassium, and 3.1 mg zinc (Table 2). The vegetarian-style diet was substantially low (1274 mg) in sodium, a nutrient to reduce [5,6]. No significant differences were noted in the estimated mean intakes of calcium, magnesium, and iron. After adjusting for the demographic characteristics, these differences continued to be significant.

When nutrient densities (amounts of micronutrients present per 1000 kilocalories of energy intake) of the two diets were compared, some of the significant differences noted in Table 2 disappeared (Table 3). The vegetarian-style diet was significantly more nutrient dense with respect to dietary fiber, calcium, magnesium, potassium, and iron, but continued to be significantly lower in protein, cholesterol, vitamin B12, zinc, and sodium than the nonvegetarian diet. 

### 3.3. Food Pattern Food Group Intakes

Table 4 includes the estimated mean intakes of Dietary Guidelines food pattern groups. The mean intakes of the adults consuming a vegetarian-style diet were significantly high in whole grains, legumes, and nuts, seeds, and soy products, and significantly low in solid fats. There were no differences in the mean intakes of total fruit, fruit juice, total vegetables, eggs, fluid milk, and cheese between the two groups. Adjusting for demographic variables removed the apparent differences in the unadjusted intact fruit and added sugars intakes.

### 3.4. Body Measurements and Serum Cholesterol

In both males and females, no significant differences were noted between the two dietary pattern groups, in their mean BMI, percentages overweight adults, and mean waist circumference (Table 5). The mean BMI of both males and females were in the overweight category. More than 70% of males and 65% of females were overweight. 

About 96% (*N* = 9642) of the adults in the study had serum total cholesterol and direct HDL-cholesterol values. No significant differences were noted in the mean serum total cholesterol levels or the direct HDL-cholesterol levels between the two diet groups. Both groups had 192 (0.8) mg/dL total cholesterol and 54 (0.4) mg/dL direct HDL-cholesterol. More than 50 percent of adults in the sample did not have serum LDL-cholesterol and serum triglyceride values, and hence, these two parameters are not reported.

## 4. Discussion

This study provides a snapshot of food and nutrient intakes of free-living adults in the United States, who ate a vegetarian-style diet. The study findings showed women are more likely to eat a vegetarian diet than men. Alles et al. [34] using the NutriNet-Sante Study noted that vegetarians were more likely to be women.

The vegetarian diet was 419 kcals lower in calories. However, this finding did not translate to lower BMI or lower waist circumference for men and women. Large cohort studies show vegetarians and vegans are likely to have a lower BMI and have a lower risk for heart disease [13,35,36,37]. Findings from the GEICO multi-center plant-based nutrition intervention study [38] and the BROAD study, a randomized controlled trial using a whole-food, plant-based diet, included weight loss and improvement in BMI and a reduction in LDL-cholesterol in the plant-based diet group [39]. 

There may be a few reasons for not seeing any differences in BMI of vegetarians and nonvegetarians in this study. First, it is necessary to note that the adults, who had vegetarian-style diet on the survey day, might not be 100% vegetarians every day. Similarly, some nonvegetarians might eat a vegetarian diet on non-survey days. The current study, which is based on one-day dietary recall and may not reflect a habitual dietary pattern. Changes in body weight are reflective of long term dietary and lifestyle habits. 

Another reason for not seeing results similar to the large cohort studies could be due to the differences in the demographic characteristics of the study participants. The EPIC-Oxford and the AHS-2 study participants are more homogeneous, and the NHANES samples represent diverse demographics. The NHANES respondents are selected to be nationally representative with respect to age, gender, race-ethnicity and family income [26,27], whereas, in the two large cohort studies, participants were selected based on their dietary choices. In the EPIC-Oxford study, one-third of men and one-fourth of women were either vegetarians or vegans [40]; and in the AHS-2, 42 percent were vegetarians [41]. 

Kennedy et al. using the USDA’s nationally representative dietary survey, CSFII 1994–1996 data, found that vegetarian males and females had significantly lower BMI than the respective nonvegetarians; and the vegetarian’s diet was 464- calories less than the nonvegetarian diet [7]. The NHANES data are used to track the changes in the percentages of U.S. population who are overweight or obese. In 1995, less than two-thirds of the adults were overweight as compared to the 72 percent in 2015–2016 [42]. Because about three-fourths of the adults in this study were overweight to start with, their dietary patterns, very likely, did not have an impact on their BMI.

An 18-week, multi-center randomized control trial of plants-based nutrition program conducted by US Insurance GEICO found a significant reduction in the intakes of energy, total fat, saturated fat, monounsaturated fat, cholesterol, and protein, and increases in the intakes of carbohydrate and in the intervention group compared with the control group [38]. Similar to the GEICO study findings, Davey et al. and Sobiecki et al. in the EPIC-Oxford study observed that vegetarian diets were lower in calories, saturated fat, and cholesterol, and higher in dietary fiber, and magnesium [22,40]. In AHS-2 vegetarians had higher dietary fiber and lower saturated fat intakes than nonvegetarians. Vegetarians had fruit, vegetables, soy, and whole grains than nonvegetarians [42,43]. The current study on free-living adults found similar pattern in the macronutrient intakes of vegetarians. Higher whole grains, legumes, soy products, nuts and seeds in the vegetarian diet resulted in higher dietary fiber intakes of vegetarians. Dietary fiber, especially whole grains fiber have been shown to reduce energy intake by promoting satiation and satiety [40,44,45]. 

The vegetarian diet was lower in protein than the nonvegetarian diet in the current study. However, no significant differences were noted in the intakes of animal protein foods fluid milk, cheese, and eggs between the vegetarian and nonvegetarian groups. Most of the difference in the protein intakes came from the meat, poultry, and seafood intakes by the nonvegetarians. Other studies have also noted low protein intakes in vegetarian diets [25,38]. About 5% of men and 8% of women in the United States did not meet the EAR for protein in 2013–2016 [46]. Protein could be one of the important nutrient that may be limiting in the vegetarian’s diet. Vegetarian may increase their protein intake by including more of whole grains, soy products, legumes, nuts, and seeds. Lacto-ovo-vegetarians may consume low-fat or nonfat dairy products and eggs to increase the amount and quality of protein. 

The DGA 2015–2020 provide food-based recommendations to meet the nutrient requirements by eating a nutrient-dense diet. In general, the vegetarian-style diet was more nutrient dense than the nonvegetarian diet (Table 3). However, the vegetarians’ mean intakes of vitamins B6, vitamin 12, vitamin D, phosphorus, potassium, and zinc were lower than that of nonvegetarians (Table 2). Similar results have been observed in other studies [22,38]. Unlike the GEICO study [38] and the EPIC-Oxford study [22], where the vegetarian diets were lower in calcium, this study did not find difference in the calcium intakes of vegetarians and nonvegetarians. Other studies have noted that the vegetarian diets may be limiting in vitamin B12 and zinc [22,38,41,47]. In the current study, the mean intakes of vitamin B12 adjusted for demographic characteristics was 3.5 µg and was above the EAR of 2 µg, and the mean intake of zinc was 8.7 mg and was above the EAR for women, and was below the 9.4 mg, EAR for men. The usual intake analyses of NHANES 2013–2016 data, which included nutrients from foods and beverages and excluded dietary supplements, showed, less than 3 percent of American males and females do not meet the EAR for zinc and less than 3 percent of males and less than 8 percent of females do not meet EAR for vitamin B12 [46]. It is likely, most vegetarians meet zinc and vitamin B12 recommendations. Where necessary, appropriate dietary supplements may be recommended.

Another notable finding include that the vegetarian-style diet was 1274 mg lower in sodium. Their lower sodium intake may be attributed to eating more healthful foods such as whole grains, whole or intact fruit, legumes, nuts, and seeds. The vegetarians’ solid fats intake was lower, indicating a lower consumption of fried foods such as snacks that may contain added salt. About 29% of individuals 18 years and older in the United States have hypertension [48] and a vegetarian-style dietary pattern may help reduce sodium intakes of hypertensive individuals. 

A meta-analysis conducted by Yokoyama et al. that included 30 observational studies and 19 clinical trials showed that plant-based diet were associated with decreased total cholesterol, low-density lipoprotein cholesterol, and high-density lipoprotein cholesterol [21]. Wang et al.’s meta-analysis showed similar reductions in the three types of cholesterols [20]. The current study did not see differences in the total and high-density lipoprotein cholesterols between the two diet groups. A reason may be that the current study is cross-sectional and the vegetarians in this study might not be habitual vegetarians, whereas, the two meta-analyses included intervention studies and long-term studies that were designed to study specific dietary patterns and outcomes.

One of the strengths of the study is that it did not use adults’ perception of whether they are vegetarians or not, but instead, used their self-reported dietary intakes to group them into vegetarians or nonvegetarian. Another strength being, this study used nationally representative dietary data, and used DGA 2015–2020 definitions to classify the vegetarian-style diet, modelled as lacto-ovo-vegetarian dietary pattern, and estimated intakes of DGA food pattern food groups, and thus making the study findings useful to inform national nutrition policy. Although long-term cohort studies are better suited to study the changes in the dietary patterns, the NHANES being national surveys, the study results are valuable in providing national level information on food and nutrient intakes that inform dietary guidance and public health policy [5,49], and for monitoring changes over time [50].

Limitations include the use of cross-sectional dietary data collected using a 24-h recall method, which is subject to measurement errors [51]. After the integration of CSFII with NHANES in 2002, dietary intake data have been collected through a 24-h recall using standardized AMPM method developed by USDA [52]. The accuracy of AMPM has been shown by the 24-h urinary sodium data from the AMPM Validation Study [53]. The Food and Nutrient Databases for Dietary Studies (FNDDS) and the Food Patterns Equivalent Databases (FPED) are updated for each 2-year NHANES survey cycle, to reflect nutrient and food pattern profiles of the foods and beverages in the food supply. Also, the 24-h dietary recalls require short-term memory and do not require a high level of literacy of the respondents [54] since it is administered by a trained dietary interviewer, and therefore can be used to study all demographic groups.

It should be noted that eating a vegetarian-style diet does not necessarily mean that all of the food choices made are nutritious, but might also include less nutritious foods and beverages. This study did not look at the food and beverage intakes and their nutrient contributions to the vegetarian and non-vegetarian diets. Future studies on the intakes of foods such as breakfast cereals, vegetables and vegetable salads, soy-based foods and legumes, fruits and fruit juices, snacks, fried foods, nuts and seeds, mixed dishes, different beverages types would help identify the similarities and differences in the dietary practices between the two groups, and the areas for dietary improvement. Examination of the use of dietary supplements would be useful to identify under or over consumed nutrients.

## 5. Conclusions

The diet of adults consuming DGA 2015–2020 defined vegetarian-style pattern (modelled as lacto-ovo-vegetarian diet), is mostly composed of plant-based foods along with dairy and eggs was characterized by low calories, total fat, saturated fat, and sodium. Because of the association between calories and body weight; saturated fat, LDL-cholesterol, and heart disease; and sodium and hypertension, adopting vegetarian-style eating pattern will help control diet-related chronic health conditions such as obesity, hypertension, and heart disease, to name a few. Intake of protein and limiting micronutrients in vegetarian diets may be increased by including fortified cereals, whole grains, low fat or nonfat dairy products, soy products, legumes, nuts and seeds. If necessary, micronutrient supplements may be advocated.

In spite of the substantial difference in the energy intakes, a lack of difference in the BMI of vegetarians and nonvegetarians indicates other lifestyle characteristics might play a role. Habitual vegetarians who are overweight may need dietary and lifestyle interventions to attain a healthy body weight. Dietary guidance may be provided to the clients on shopping for nutrient-dense foods and beverages and on the preparation of healthier foods that are mostly plant-based.

## Figures and Tables

**Table 1:** Demographic characteristics of adults 20 years over, grouped by vegetarian-style dietary pattern, WWEIA, NHANES ^1^ 2013–2016, day 1 data.

Demographic Characteristics	Percent Eating a Vegetarian-Style Diet (SE)	Dependent Variable = Vegetarian-Style Dietary Pattern. Odds Ratio (99% CI)
All adults	7.0	
Sex:		
Males	5.5 (0.4) ^a^	1.0
Females	8.4 (0.6) ^b^	1.6 * (1.13–2.25)
Race-ethnicity:		
Non-Hispanic white	7.1 (0.5) ^c^	1.0
Non-Hispanic black	3.4 (0.5) ^d^	0.45 ** (0.28–0.72)
Non-Hispanic Asian	14.5 (1.4) ^e^	2.22 *** (1.57–3.14)
Hispanic	6.7 (0.7) ^c^	0.94 (0.68–1.32)

^1^ What We Eat in America (WWEIA), National Health and Nutrition Examination Survey (NHANES). Comparisons were made within the same demographic group. ^a–e^ Percentages with same superscripts are not significantly different from each other at *p* < 0.01. *, **, *** significantly different from the reference group at *p* < 0.01. SE indicates standard error. CI indicates confidence interval. Age and percentage of poverty level as a categorical variable in the logistic model were not significant at *p* < 0.01, and hence are not in Table 1.

**Table 2:** Mean intakes of energy, and selected nutrients by adults 20 years over, grouped by dietary patterns, WWEIA, NHANES ^1^ 2013–2016, day 1 data.

Variables	Unadjusted Means (SE)		Means (99% CI) Adjusted for Sex, as, Race-Ethnicity, and Poverty Level	
	Vegetarian-Style Dietary Pattern (*N* = 675)	Nonvegetarian Dietary Pattern (*N* = 9389)	Vegetarian-Style Dietary Pattern	Nonvegetarian Dietary Pattern
Energy (kcal)	1733 (60) ^a^	2152 (14) ^b^	1811 (1649–1972) ^c^	2146 (2116–2176) ^d^
Total fat (g)	66 (2.8) ^a^	85 (0.7) ^b^	69 (61–77) ^c^	85 (83–86) ^d^
Saturated fat (g)	21 (0.9) ^a^	28 (0.3) ^b^	22 (20–25) ^c^	28 (27–29) ^d^
Monounsaturated fat (g)	23.2 (1.1) ^a^	29.6 (0.2) ^b^	24.4 (21.3–27.6) ^c^	29.6 (29.0–30.0) ^d^
Polyunsaturated fat (g)	15.3 (0.8) ^a^	19.7 (0.2) ^b^	16.0 (13.9–18.0) ^c^	19.7 (19.1–20.2) ^d^
Cholesterol (mg)	166 (13) ^a^	306 (4) ^b^	178 (139–216) ^c^	306 (297–314) ^d^
Carbohydrate (g)	231 (8) ^a^	250 (2) ^a^	239 (216–260) ^c^	249 (245–253) ^d^
Dietary fiber (g)	20 (1) ^a^	17 (0.3) ^b^	20 (18–23) ^c^	17 (16–18) ^d^
Protein (g)	52 (2) ^a^	85 (1) ^b^	54 (48–61) ^c^	85 (83–87) ^d^
Vitamin B6 (mg)	1.7 (0.1) ^a^	2.2 (0.03) ^b^	1.8 (1.5–2.0) ^c^	2.2 (2.1–2.3) ^d^
Vitamin B12 (µg)	3.2 (0.18) ^a^	5.1(0.8) ^b^	3.5 (2.9–4.0) ^c^	5.1 (4.9–5.3) ^d^
Vitamin A (RAE)	687 (35) ^a^	629 (9) ^a^	692 (596–789) ^c^	629 (604–653) ^c^
Vitamin D (D2 + D3) (µg)	3.9 (0.2) ^a^	4.8 (0.1) ^b^	4.0 (3.3–4.6) ^c^	4.8 (4.6–5.1) ^d^
Calcium (mg)	951(32) ^a^	958 (11) ^a^	975 (889–1061) ^c^	956 (931–980) ^c^
Phosphorus (mg)	1136 (42) ^a^	1411(13) ^b^	1177 (1061–1294) ^c^	1408 (1379–1438) ^d^
Magnesium (mg)	310 (10) ^a^	306 (3) ^a^	315 (286–344) ^c^	305 (298–313) ^c^
Potassium (mg)	2291(67) ^a^	2672 (26) ^b^	2343 (2163–2524) ^c^	2668 (2606–2730) ^d^
Iron (mg)	13.5 (0.6) ^a^	14.3 (0.1) ^a^	13.9 (12.2–15.6) ^c^	14.3 (14.0–14.6) ^c^
Zinc (mg)	8.3(0.4) ^a^	11.4 (0.1) ^b^	8.7 (7.6–9.7) ^c^	11.4 (11.1–11.7) ^d^
Sodium (mg)	2347(80) ^a^	3621(27) ^b^	2426 (2210–2641) ^c^	3615 (3544–3686) ^d^

^1^ What We Eat in America, National Health and Nutrition Examination Survey. ^a,b^ Means with different superscripts are significantly different from each other at *p* < 0.01. ^c,d^ Adjusted means with different superscripts are significantly different from each other at *p* < 0.01. SE indicates standard error. CI indicates confidence interval.

**Table 3:** Mean intakes of selected nutrients per 1000 kilocalories by adults 20 years over, grouped by dietary patterns, WWEIA, NHANES ^1^ 2013–2016, day 1 data.

Variables	Unadjusted Means (SE) per 1000 Kilocalories	
	Vegetarian-Style Dietary Pattern	Nonvegetarian Dietary Pattern
Total fat per 1000 kcals (g)	37.0 (0.8) ^a^	38.9 (0.2) ^a^
Saturated fat per 1000 kcals (g)	11.9 (0.3) ^a^	12.6 (0.1) ^a^
Cholesterol per 1000 kcals (mg)	103 (8.9) ^a^	145 (1.3) ^b^
Carbohydrate per 1000 kcals (g)	136 (2.0) ^a^	117 (0.5) ^a^
Dietary fiber per 1000 kcals (g)	11.9 (0.4) ^a^	8.2 (0.1) ^b^
Protein per 1000 kcals (g)	30.6 (0.9) ^a^	40.9 (0.3) ^b^
Vitamin B6 per 1000 kcals (mg)	1.0 (0.05) ^a^	1.1 (0.01) ^a^
Vitamin B12 per 1000 kcals (µg)	2.0 (0.11) ^a^	2.4 (0.01) ^b^
Calcium per 1000 kcals (mg)	590 (23) ^a^	459 (4.2) ^b^
Phosphorus per 1000 kcals (mg)	666 (16.3) ^a^	669 (4.7) ^a^
Magnesium per 1000 kcals (mg)	188 (4.5) ^a^	148 (1.3) ^b^
Potassium per 1000 kcals (mg)	1420 (36) ^a^	1300 (11) ^b^
Iron per 1000 kcals (mg)	8.0 (0.3) ^a^	6.9 (0.1) ^b^
Zinc per 1000 kcals (mg)	4.9 (0.2) ^a^	5.4 (0.1) ^a^
Sodium per 1000 kcals (mg)	1451(60) ^a^	1727 (10) ^b^

^1^ What We Eat in America, National Health and Nutrition Examination Survey. ^a,b^ Means with different superscripts are significantly different from each other at *p* < 0.01. SE indicates standard error.

**Table 4:** Mean intakes of USDA Food Patterns groups by adults 20 years over, grouped by vegetarian dietary pattern, WWEIA, NHANES ^1^ 2013–2016, day 1 data.

Variables	Unadjusted Means (SE)		Means Adjusted for Sex, Race-Ethnicity, Poverty Level, and Age (99% CI)	
	Vegetarian-Style Dietary Pattern	Nonvegetarian Dietary Pattern	Vegetarian-Style Dietary Pattern	Nonvegetarian Dietary Pattern
Total grains (oz. eq. ^2^)	6.0 (0.3) ^a^	6.5 (0.05) ^a^	6.2 (5.5–6.8) ^c^	6.5 (6.4–6.6) ^c^
Refined grains (oz. eq.)	4.7 (0.2) ^a^	5.6 (0.05) ^b^	4.9 (4.3–5.4) ^c^	5.6 (5.5–5.7) ^d^
Whole grains (oz. eq.)	1.3 (0.1) ^a^	0.9 (0.03) ^b^	1.3 (1.0–1.6) ^c^	0.9 (0.8–0.94) ^d^
Total vegetables, excluding legumes (cup eq.)	1.5 (0.1) ^a^	1.5 (0.03) ^a^	1.46 (1.24–1.68) ^c^	1.54 (1.47–1.60) ^c^
Legumes as vegetables (cup eq.)	0.2 (0.02) ^a^	0.1(0.01) ^b^	0.20 (0.15–0.25) ^c^	0.11 (0.10–0.13) ^d^
Nuts, seeds, soy products (oz. eq.)	1.7 (0.2) ^a^	0.9 (0.03) ^b^	1.7 (1.1–2.2) ^c^	0.9 (0.8–0.94) ^d^
Total fruit (cup eq. ^3^)	1.1 (0.1) ^a^	0.9 (0.03) ^a^	1.1 (0.8–1.3) ^c^	0.9 (0.8–1.0) ^c^
Intact/whole fruit (cup eq.)	0.9 (0.07) ^a^	0.7 (0.02) ^b^	0.85 (0.66–1.05) ^c^	0.67 (0.61–0.73) ^c^
Fruit juice, 100% (cup eq.)	0.2 (0.02) ^a^	0.2 (0.01) ^a^	0.21 (0.15–0.27) ^c^	0.24 (0.22–0.26) ^c^
Total dairy (cup eq.)	1.7 (0.08) ^a^	1.6 (0.03) ^a^	1.7 (1.8–1.9) ^c^	1.55 (1.49–1.61) ^c^
Fluid milk (cup eq.)	0.7 (0.05) ^a^	0.7 (0.02) ^a^	0.8 (0.6–0.9) ^c^	0.7 (0.6–0.7) ^c^
Cheese (cup eq.)	0.7 (0.05) ^a^	0.8 (0.02) ^a^	0.8 (0.6–0.9) ^c^	0.8 (0.7–0.8) ^c^
Eggs (oz. eq.)	0.6 (0.06) ^a^	0.6 (0.02) ^a^	0.6 (0.4–0.8) ^c^	0.6 (0.5–0.6) ^c^
Total meat, poultry, and seafood (oz. eq.)	--	5.2 (0.1)	--	5.2 (5.0–5.4)
Solid fats (grams)	27 (1.4) ^a^	36 (0.4) ^b^	29 (25–33) ^c^	36 (35–37) ^d^
Oils (grams)	26 (1.6) ^a^	28 (0.4) ^a^	26 (22–31) ^c^	28 (27–29) ^c^
Added sugars (tsp. eq. ^4^)	14 (0.7) ^a^	17 (0.3) ^b^	15 (12.9–17.1) ^c^	17 (16.4–17.7) ^c^

^1^ What We Eat in America, National Health and Nutrition Examination Survey. ^2^ oz. eq. = ounce equivalents; ^3^ cup eq. = cup equivalents; ^4^ tsp. eq. = teaspoon equivalents. ^a,b^ Unadjusted means with different superscripts are significantly different from each other at *p* < 0.01. ^c,d^ Adjusted means with different superscripts are significantly different from each other at *p* < 0.01. SE indicates standard error. CI indicates confidence interval.

**Table 5:** Mean Body Max Index and waist circumference of adults 20 years over, grouped by dietary patterns, NHANES ^1^ 2013–2016.

Variables	Vegetarian-Style Dietary Pattern	Nonvegetarian Dietary Pattern
**Males**		
Body Mass Index (kg/m^2^)	28.0 (0.7) ^a^	29.0 (0.2) ^a^
Waist circumference (cm)	100.3 (1.7) ^a^	102.2 (0.4) ^a^
Percent overweight	70.5 (3.5) ^a^	73.9 (1.0) ^a^
**Females**		
Body Mass Index (kg/m^2^)	28.3 (0.5) ^a^	29.7 (0.2) ^a^
Waist circumference (cm)	95.5 (1.2) ^a^	98.1 (0.5) ^a^
Percent overweight	64.2 (2.8) ^a^	68.7 (1.2) ^a^

^1^ National Health and Nutrition Examination Survey. ^a^ Means with different superscripts are significantly different from each other at *p* < 0.01. ( ) standard error.
